# DNA Methylation as a Diagnostic, Prognostic, and Predictive Biomarker in Head and Neck Cancer

**DOI:** 10.3390/ijms24032996

**Published:** 2023-02-03

**Authors:** Galateia Liouta, Maria Adamaki, Antonis Tsintarakis, Panagiotis Zoumpourlis, Anastasia Liouta, Sofia Agelaki, Vassilis Zoumpourlis

**Affiliations:** 1 Biomedical Applications Unit, Institute of Chemical Biology, National Hellenic Research Foundation (NHRF), 11635 Athens, Greece; 2 Department of Medical Oncology, University General Hospital of Heraklion, Vassilika Vouton, 71110 Heraklion, Greece; 3 Laboratory of Translational Oncology, School of Medicine, University of Crete, Vassilika Vouton, 71003 Heraklion, Greece

**Keywords:** head and neck cancer, DNA methylation, diagnostic biomarkers, prognostic biomarkers, predictive biomarkers

## Abstract

Head and neck squamous cell carcinoma (HNSCC) is a term collectively used to describe all cancers that develop in the oral and nasal cavities, the paranasal sinuses, the salivary glands, the pharynx, and the larynx. The majority (75%) of all newly diagnosed cases are observed in patients with locally advanced and aggressive disease, associated with significant relapse rates (30%) and poor prognostic outcomes, despite advances in multimodal treatment. Consequently, there is an unmet need for the identification and application of tools that would enable diagnosis at the earliest possible stage, accurately predict prognostic outcomes, contribute to the timely detection of relapses, and aid in the decision for therapy selection. Recent evidence suggests that DNA methylation can alter the expression of genes in a way that it favors tumorigenesis and tumor progression in HNSCC, and therefore represents a potential source for biomarker identification. This study summarizes the current knowledge on how abnormally methylated DNA profiles in HNSCC patients may contribute to the pathogenesis of HNSCC and designate the methylation patterns that have the potential to constitute clinically valuable biomarkers for achieving significant advances in the management of the disease and for improving survival outcomes in these patients.

## 1. Introduction

Head and neck squamous cell carcinoma (HNSCC) is a general term that includes all cancers that develop in the oral and nasal cavities, the paranasal sinuses, the salivary glands, the pharynx, and the larynx [1]. Most of these cancers are initiated in the squamous cells that line the moist surfaces inside the head and neck (hence the term head and neck squamous cell carcinoma), and are characterized by heterogeneity in their phenotypic, clinical, and biological features [2,3,4,5]. According to the Globocan online database, in 2020, it was estimated that there were 931,931 new cases of HNSCC and 467,125 related deaths worldwide [6]. The majority of patients who will be diagnosed at an early stage can be cured [4]. However, patients with locally advanced and aggressive disease, who account for 75% of the newly diagnosed cases, are likely to experience relapse and have a 5-year overall survival (OS) rate of 50%, despite advances in surgical treatment, radiotherapy, and chemotherapy [4,5]. Consequently, there is a need for the identification and application of tools with high sensitivity and specificity that would enable diagnosis at the earliest possible stage, inform clinicians of the possible prognosis of patients, contribute to the early detection of relapses, and provide information on the progression of the disease after the application of specific treatments [7].

Common predisposing factors are tobacco use and alcohol consumption for HNSCC, Epstein–Barr virus (EBV) infection for nasopharyngeal carcinoma, and human papillomavirus (HPV) infection, especially subtypes 16 and 18, for oropharyngeal cancer [2,3,8,9,10,11]. Although the prevalence of both smoking and alcohol abuse has decreased in recent years, the incidence of HNSCC is increasing worldwide, and this has been attributed to the higher incidence of HPV infection [1,2,3].

One of the mechanisms by which smoking, alcohol abuse, and HPV infection increase the risk of developing HNSCC is through the induction of epigenetic changes that lead to abnormal cellular physiology [12,13]. Specifically, these are alterations at the chromosomal level that lead to changes in gene expression without altering the DNA sequence [13]. The most common of these alterations are DNA methylation, non-coding RNAs, and histone modifications [13,14]. DNA methylation, in particular, involves the addition of a methyl group from S-adenosyl methionine (SAM) to the fifth carbon of cytosine (C) to form 5-methylcytosine (5 mC), under the catalytic action of DNA methyltransferases (Dnmts) [14,15,16]. Specifically, Dnmt3a and Dnmt3b are responsible for the catalysis of the methylation of Cs whereas Dnmt1 is responsible for the transfer of the methylation pattern during cell division [16]. DNA methylation has been associated with numerous physiological cellular processes, such as transcriptional silencing, inactivation of the X chromosome, embryonic development, genomic imprinting, alterations in chromatin structure, and transposon inactivation [16,17]. Most often, methylation occurs within CpG islands, i.e., within genomic regions with a higher than usual frequency of CpG nucleotides, which are mainly located in promoter regions [14,16]. The outcome is the change in the affinity of transcription factors to target genes, as well as the mobilization of other proteins, such as methyl binding domain proteins and chromatin remodelers [14]. Notably, DNA methylation can act both inside and outside of the CpG islands [14,16]. When DNA methylation affects the promoter region, this usually results in gene silencing [14,16]. In contrast, when DNA methylation affects the main region of the gene, the result is usually an increase in gene expression [17]. The various patterns of methylation are both inheritable and dynamic, in that they are reshaped during the reprogramming phases in an organism’s life cycle [16]. Thus, both smoking and alcohol have been associated with hypomethylation in HNSCC, while HPV-positive (HPV+) tumors show higher levels of methylation than HPV-negative (HPV−) tumors [14]. Indeed, in HNSCC, in addition to the tumor suppressor genes that are downregulated due to cancer-specific methylation of their promoter, such as *PAX1* and *PAX5*, tumor suppressor genes that are inactivated by both promoter methylation and somatic mutations (*GABRB3*, *HOXC12*, *PARP15*, *SLCO4C1*, *CDKN2A*, *PAX1*, *PIK3AP1*, *HOXC6*, *PLCB1*, and *ZIC4*) have also been recognized [18].

A biomarker is a biological finding that stands in for and optimally forecasts a clinically related outcome or an intermediate result that is more difficult to detect [19]. It is a specific characteristic that is measured as an indicator of the normal biological procedures, pathological mechanisms or responses to an exposure or interference [20]. This study summarizes the current knowledge on how abnormally methylated DNA profiles in HNSCC patients may contribute to the pathogenesis of HNSCC and designate the methylation patterns that have the potential to constitute clinically valuable biomarkers for achieving significant advances in managing the disease and for improving survival outcomes in these patients.

## 2. DNA Methylation Biomarkers with Diagnostic Potential

A diagnostic biomarker is used to identify or verify the occurrence of a disease or a situation of interest, or to designate a specific disease subtype [20].

MicroRNAs (miRNAs) represent small, non-coding RNAs that are involved in gene expression either through repression of translation or through direct degradation of the target mRNA and are also actively implicated in oncogenesis [21]. The methylation status of three promoter sites of microRNA 9 (miRNA 9), namely miR-9-1, miR-9-2, and miR-9-3, are perhaps the most widely studied microRNAs in patients with HNSCC [21]. Oral and oropharyngeal carcinomas are characterized by higher miR-9-1 and miR-9-3 methylation levels, as compared to laryngeal carcinomas, while miR-9 expression appears to be reduced [21]. Consequently, the methylation patterns of miR-9-1 and miR-9-3 are regarded as sensitive and highly specific for the diagnosis of HNSCC, especially oral and oropharyngeal carcinomas [21].

The *EDNRB* gene encodes the B-type endothelin receptor (G protein-coupled receptor) that activates a phosphatidylinositol-calcium second messenger system, while *DCC*, a tumor suppressor gene, encodes a transmembrane protein with structural homology to NCAM, which is involved in the differentiation of epithelial and neuronal cells [22]. The promoters of both genes have been found to be hypermethylated in 40% of saliva samples from patients with oral cavity cancer (OCC) and precancerous lesions [22]. Thus, hypermethylation of *DCC* and *EDNRB* has been associated with malignant histopathological diagnosis, independent of other factors such as age, smoking, and alcohol consumption; this further suggests that these genes can be used as individual biomarkers of malignant transformation for the screening of high-risk patients, as well as for the identification of patients who might appear to be at low risk during physical examination, but are categorized as high risk based on the salivary methylation biomarkers [22].

The mediator complex subunit 15 gene (*MED15/PCQAP*) encodes a cofactor that contributes greatly to the modulation of transcription initiation in the promoters of many genes [23]. Two CpG dinucleotide clusters (at the 5′ or 3′ end) of this gene have been identified to be methylated in HNSCC tumors in patients who have been smokers, but not in normal tissue samples derived from the same subjects, with subsequent validation of the findings in saliva samples [23]. It has therefore been concluded that these CpG methylation sites may be used as potential non-invasive biomarkers for HNSCC detection [23]. Specifically, the 5′-CpG site has emerged as a stronger diagnostic biomarker than the 3′-CpG site, with the DNA methylation levels of both sites being comparatively lower in saliva samples of HPV+ patients as compared to HPV− patients [23]. Moreover, it has been reported that the differential methylation of *ZNF14*, *ZNF160,* and *ZNF420*, all of which are members of the zinc finger gene family, constitutes an important biomarker of HNSCC identification, exhibiting 100 % specificity in primary tissue and saliva samples [24].

Other genes with abnormal methylation patterns and consequently with differential expression patterns in HNSCC tissues have been detected through bioinformatics analysis [25]. Two hypermethylated genes with concomitant low expression, *FAM135B* and *ZNF610*, and two hypomethylated genes with concomitant high expression, *HOXA9* and *DCC*, have been identified, and their diagnostic utility has been validated through ROC curve analysis [25]. In contrast to these observations, the *HOXA9* promoter appears to be characterized by considerably higher methylation levels in pathological tissues, as compared to normal controls, and may therefore serve as an early diagnostic biomarker in patients with HNSCC [26]. Nonetheless, a statistically significant difference in *HOXA9* methylation seems to exist between men and women with HNSCC [26].

In HPV+ oropharyngeal squamous cell carcinoma (OPSCC), 20 differentially methylated DNA regions (DMRs) have been identified in the following genes: *KCNA3*, *EMBP1*, *CCDC181*, *DPP4*, *ITGA4*, *BEND4*, *CTNND2*, *ELMO1*, *SFMBT2*, *C1QL3*, *MIR129-2*, *ATP5EP2*, *OR6S1*, *NID2*, *HOXB4*, *ZNF439*, *ZNF93*, *VSTM2B*, *ZNF137P*, and *ZNF773* [27]. The methylation levels in HPV+ OPSCC are remarkably higher than those in normal samples and non-HPV-related HNSCC, and as such, these 20 DMRs have been suggested as potential diagnostic biomarkers in patients with HPV+ OPSCC [27].

Prominin 1 (*PROM1*) encodes a pentaspan membrane glycoprotein, often expressed on adult stem cells [28]. The *PROM1* promoter appears to be hypermethylated in HNSCC tissues, as compared to normal head and neck tissues, while increasing methylation levels are negatively associated with *PROM1* gene expression, with the highest methylation levels observed in smokers and elderly patients [28]. Overall, the methylation status of *PROM1* may serve as a valuable biomarker for the early diagnosis of HNSCC [28].

The collagen triple helix repeat containing 1 (*CTHRC1*) gene encodes an extracellular matrix protein that acts as a modulator of the tumor microenvironment, and appears to be overexpressed in HNSCC tissues, as compared to healthy tissues, due to promoter hypomethylation [29]. Furthermore, plasminogen activator urokinase (PLAU) overexpression may be an independent diagnostic and prognostic biomarker in HNSCC [30]. PLAU is a protease involved in numerous different signaling pathways, including apoptosis, epithelial-mesenchymal transition (EMT), and Ras/MAPK; hypomethylation of *PLAU* gene as well as hypomethylation and subsequent downregulation of miR-23b-3p, a microRNA that targets *PLAU*, may be responsible for the overexpression and the oncogenic role of *PLAU* in HNSCC [30].

Other genes with potential diagnostic utility in HNSCC include methylenetetrahydrofolate dehydrogenase 1 (*MTHFD1L*), the opioid receptor genes *OPRL1* (opioid-related nociceptin receptor 1) and *OPRM1* (opioid receptor mu 1), and the xenotropic and multimodal retrovirus receptor 1 (*XPR1*). *MTHFD1L* expression levels have been found to be significantly higher in 24 subtypes of HNSCC, compared to normal controls, and to be accompanied by promoter hypomethylation [31]. Similarly, in plasma liquid biopsy samples from patients with OCC, *OPRL1* and *OPRM1* genes appear to be highly methylated as compared to normal tissue derived from the same patients [32]. Last but not least, XPR1, a cell surface receptor for certain types of murine leukemia viruses, exhibits markedly increased expression in HNSCC tissues, as compared to healthy controls, while promoter methylation is significantly lower than that in healthy controls [33].

Table 1 includes a list of DNA methylation biomarkers with diagnostic potential [21,22,23,24,25,26,27,28,29,30,31,32,33].

## 3. DNA Methylation Biomarkers with Prognostic Potential

A prognostic biomarker can give us information on the probability of a clinical condition, the possibility of relapse or disease progression [20]. An extensive list of methylated genes and proteins with prognostic potential is discussed below.

The p16 and p14 proteins, acting as inhibitors of cell cycle progression, are derived from two different splice variants, *P16INK4A* and *p14ARF*, of the same gene, namely *CDKN2A* [33]. Abnormally high *P16INK4A* and *p14ARF* methylation levels have been observed in OSCC samples, and this hypermethylation is considered an early event in the carcinogenesis process, as it is evident both in precancerous lesions and peri-tumor tissues [34]. Notably, *p16* promoter methylation has also been validated as a prognostic biomarker of the progression of precancerous lesions, advanced disease, locally recurring disease, and disease-specific survival in OSCC [34].

In addition, the abnormal methylation patterns of somatostatin (*SST*) and somatostatin receptor type 1 (*SSTR1*) genes and their significance as prognostic biomarkers have been evaluated in HNSCC [35]. SST hypermethylation, with subsequent decrease in gene expression, has been observed in 81% of HNSCC samples and has been linked to tumor extent, disease stage, galanin type 2 receptor (*GALR2*) methylation, and tachykinin-1 (*TAC1*) methylation [35]. *SSTR1* hypermethylation has been detected in 64% of HNSCC samples and has also been associated with tumor extent, disease stage, *SST* hypermethylation, and increased expression of galanin (*GAL*), *GALR2*, *TAC1*, and tachykinin type 1 receptor (*TACR1*) [35]. Interestingly, concomitant promoter hypermethylation of galanin and *SSTR1* has been associated with a decrease in disease-free survival (DFS); synchronous promoter hypermethylation of *SST* and *SSTR1* has been associated with disease recurrence following surgical resection, in addition to being linked to a decrease in DFS [35]. It has therefore been concluded that the high methylation patterns in these genes can be used as prognostic biomarkers that can help to identify high-risk HNSCC patients who may need adjuvant therapy and tight monitoring following surgical removal of the primary tumor [35]. Promoter hypermethylation of galanin (*GAL*) and galanin receptor 1 and 2 (*GALR1/2*) has shown a positive association with relapse and the female gender in OCC, and a positive correlation with the presence of HPV infection and relapse in patients with OSCC [36]. Nonetheless, increased methylation of the *GAL*, *GALR1* or *GALR2* promoters has been linked to a statistically significant reduction in DFS in OCC and OPCC patients, while the highest association between high methylation status and poor survival has been noted in HPV− OPCC patients [36]. The methylation patterns of *GAL* and *GALR1/2* may therefore constitute important prognostic biomarkers in patients with HNSCC [36].

Genome-wide methylation analysis in oral tongue squamous cell carcinoma (OTSCC) has identified 16 differentially methylated areas that have been validated through The Cancer Genome Atlas (TCGA) database [37]. Specifically, hypermethylation of *MIR10B* has been associated with reduced expression of two of its target genes, *NR4A3* and *BCL2L11*, and with better DFS [37]. At the same time, the differential methylation status of *FUT3*, *TRIM5*, *TSPAN7*, *MAP3K8*, *RPS6KA2*, *SLC9A9*, and *NPAS3* genes has been shown to be predictive of nodal state, tumor stage, HPV status, and prognostic outcome [37]. Similarly, a signature that has been associated with significantly better survival in HPV+ HNSCC samples includes promoter hypermethylation of *GATA4*, *GRIA4*, and *IRX4* genes, and promoter hypomethylation of *ALDH1A2* and *OSR2* genes [24]. *PDCD1* hypermethylation has been strongly related to shorter overall survival (OS) following surgical resection and a strong correlation has been demonstrated with the presence of HPV, female gender, and smoking, while a negative correlation has been found with p16 as a surrogate marker for HPV-associated HNSCC [38]. Moreover, the frequency of the *PDCD1* hypermethylated motif has been remarkably higher in OSCC [38].

In contrast, analysis of the methylation status in genes that are associated with HPV− HNSCC has reported the following: (i) *COL1A2* and *VEGFR1* methylation is associated with reduced survival and a higher likelihood of relapse in patients with hypopharyngeal cancer; (ii) the methylation patterns of *p16* and *COL1A2* are independent prognostic factors of worse survival in laryngeal cancer, with *p16* promoter methylation signifying a higher risk of recurrence; (iii) *DAPK*, *TAC1*, *GALR1*, *NPY1R*, *SSTR1*, and *VEGFR3* hypermethylation is associated with poor survival in oral cancer [39]. Therefore, it has been concluded that the methylation status depends on the location and histological type of the HPV− tumor, and that these methylation markers can be used to identify the patients who may benefit from adjuvant therapy following initial resection of the tumor [39].

Furthermore, the methylation levels of the homeobox gene PITX1 (exon 3) and the adjacent long transgenic non-coding RNA C5orf66-AS1 (lincRNA) are remarkably higher in the tumor tissue than in the adjacent healthy tissue in the samples of HNSCC patients, with subsequent silencing of transcriptional expression, while hypermethylation of both genes has been correlated with an increased risk of mortality [40]. In addition, methylation of *PITX1* (exon 3) and C5orf66-AS1 lincRNA has also been strongly related to tumor localization, T class, HPV− and p16-negative cancers, and tumor grade [40]. Overall, higher levels of methylation and lower levels of mRNA expression have been noted in laryngeal and oral cavity carcinomas and HPV− tumors [40]. Nonetheless, hypomethylation of C5orf66-AS1 lincRNA has been highly related to better OS outcomes [40]. Therefore, the methylation status of *PITX1* and more so of lincRNA C5orf66-AS1 may serve as an important prognostic biomarker in HNSCC, especially in HPV− disease [40].

Abnormally high methylation levels of *FAM135B* have been identified as a positive independent prognostic biomarker of OS in HNSCC patients [25], whereas a prognostic signature of four methylated genes has been associated with prognosis and survival in patients with HNSCC who either did or did not receive radiotherapy [41]. The differentially methylated genes included zinc finger protein 10 (*ZNF10*), transmembrane protease serine 12 (*TMPRSS12*), endoplasmic reticulum-Golgi intermediate compartment protein 2 (*ERGIC2*), and ring finger protein 215 (*RNF215*); hypermethylation of the first three was associated with an unfavorable prognosis while hypomethylation and high expression of *RNF125* were linked to reduced OS [41].

Interestingly, indoleamine 2,3-dioxygenase 1 (*IDO1*) methylation can be used both as a prognostic and a predictive biomarker in HNSCC [42]. Methylation of the promoter and the promoter flank region of *IDO1* results in a decrease in mRNA expression, while methylation in the main area of the gene leads to an increase in mRNA expression [42]. In HPV+ tumors, DNA methylation in the *IDO1* promoter flank region is remarkably lower, resulting in higher mRNA expression [39], whereas hypermethylation of the *IDO1* lateral promoter has been strongly related to poor OS, which highlights its potential usefulness as a prognostic biomarker [42]. In addition, the CpG sites of cg17892178 on *NID2* and of cg17378966 on *IDO1* have been identified as the best prognostic signatures of OS in OSCC [43]. Specifically, cg17892178 hypermethylation has been associated with better survival rates and lower risk scores, while the hypermethylation of cg17378966 has been linked to worse prognostic outcomes and higher risk scores [43]. At the same time, the differential methylation patterns of *AIM2*, *BST2*, *CA3*, *CCL11*, *CHRDL1*, *CXCL5*, *GPX3*, *GREM1*, *IDO1*, *MMP9*, *NID2*, and *SFRP4* have been identified as reliable biomarkers for OSCC initiation and progression, as they have been shown to be closely associated with vascular development and immunological and inflammatory pathways [43].

*HOXA9* promoter methylation is markedly higher in patients with advanced tumor stage (T), lymph node metastasis, and advanced clinical stage in patients with HNSCC, suggesting that it may be involved in the progression and metastatic potential of the disease [26]. Bioinformatics analysis has also identified a prognostic model that is based on five genes (*PAX9*, *STK33*, *GPR150*, *INSM1*, and *EPHX3*) with differential methylation patterns that can independently predict 5-year survival [44]. Specifically, patients with hypomethylation of *PAX9*, *STK33*, *GPR150*, *INSM1*, and *EPHX3* had lower survival rates and increased tumor-related mortality [44]. Specific DNA methylation sites have also been detected in these five model DMGs, which are correlated with HNSCC prognosis, including as *PAX9* cg04994761, *STK33* cg18933494, *EPHX3* cg19744936, *GPR150* cg255831351, and *G24PR* [44].

The methylation profile of ryanodine receptor 2 (RYR2) and the resulting mRNA expression motif are highly unstable in both cancer-derived samples and HNSCC cell lines [45]. During the transition from the normal mucosa to dysplastic and oncogenic transformation, there is progressive loss of *RYR2* expression that is associated with either somatic mutation or increasing DNA methylation, thereby highlighting the potential usefulness of *RYR2* hypermethylation as a prognostic biomarker of oncogenic transformation and disease progression [45]. Similarly, hypermethylation of *CD133/PROM1* has been observed in more advanced stages of HNSCC, while it also constitutes an independent prognostic factor of poor OS and relapse-free survival [28].

A study on the nuclear factor I (NFI) family of transcription factors (namely NFIA, NFIB, NFIC, and NFIX) in various cancers, including HNSCC, has revealed significantly reduced expression levels in all NFI family members in HNSCC patients, as compared to controls, and a negative correlation with DNA methylation; accordingly, decreased expression levels of *NFIA*, *NFIB*, and *NFIC* have been correlated with shorter OS [46]. Moreover, the Dickkopf1 gene (*DKK1*), which encodes a protein with inhibitory activity on the Wnt signaling pathway, appears to be overexpressed in HNSCC tissues, as compared to healthy tissues, and this is also accompanied by hypomethylation of the gene promoter [47]. Elevated *DKK1* expression has been linked to a worse prognosis both in terms of DFS and OS [47]. The SEC61 translocon gamma subunit (*SEC61G*) is characterized by promoter hypomethylation and transcriptional activation, as compared to normal tissues adjacent to the tumors in HNSCC patients, while overexpression has also been associated with DNA amplification and negatively correlated with OS [48].

In HPV+ OPC, three genes have been identified, namely Calmodulin-like 5 (*CALML5*), DnaJ heat shock protein family member C5 gamma (*DNAJC5G*), and Lymphocyte antigen 6 complex locus D (*LY6D*), whose methylation patterns have shown a positive correlation with shorter DFS, and hence constitute useful biomarkers for predicting disease recurrence [49]. Similarly, *CTHRC1* overexpression has been associated with decreased promoter methylation and significantly lower OS rates [29]. Accordingly, plasminogen activator (*PLAU*) hypermethylation has displayed a positive correlation with survival time, while hypomethylation has been linked to a higher stage and more aggressive metastatic disease [30].

The methylation and expression pattern of tumor necrosis factor receptors *OX40* (*TNFRSF4*), and *GITR* (*TNFRSF18*, *AITR*) have also been investigated in HNSCC [50]. Both *GITR* and *OX40* are located at close range on chromosome 1 p36.33. In particular, naïve higher expression levels (hypomethylation) in tumor necrosis factor receptors *OX40* (*TNFRSF4*) and *GITR* (*TNFRSF18*, *AITR*) appear to be significantly associated with improved OS in HNSCC patients, whereas higher methylation levels of CpG5-7 (downregulation from *GITR*), CpG13-21 (*GITR* promoter), intergenic CpG31-32, and CpG42 (*OX40* promoter) are highly associated with worse survival outcomes [50]. Similar results have been obtained with methylenetetrahydrofolate dehydrogenase 1 (*MTHFD1L*), a cytoplasmic enzyme involved in the formation of tetrahydrofolate (THF) within mitochondria, where high expression levels are inversely associated with promoter hypomethylation and decreased OS [31]. In contrast, abnormal CpG island hypermethylation in the *OPRL1* and *OPRM1* genes has been independently associated with aggressive clinical behavior and an increase in relapse rates, thereby signifying that these genes have a prognostic potential, in addition to their diagnostic role [32].

In oral tongue cancer (OTC), the RIPOR family member 3 (*RIPOR3)* gene, also known as *FAM65C*, has been associated with immune cell infiltration; initially, higher expression levels were detected in pathological samples as opposed to normal tissue, but later on, *RIPOR3* downregulation was shown to be an independent predictor of poor prognosis [51]. Conversely, high *RIPOR3* expression due to promoter hypomethylation has been associated with better progression-free survival (PFS) and OS [51].

In OSCC, multi-omics analysis has identified three hypomethylated genes (*CTLA4*, *GPR29*, and *TNFSF11*) that have been strongly associated with improved OS, and one hypermethylated gene (*ISL1)* that has been linked to reduced long-term survival [52]. In addition, bioinformatics analysis on the xenotropic and multimodal retrovirus receptor 1 (*XPR1*) gene in HNSCC patients has revealed a positive association between overexpression and reduced OS, DFS, and progression-free interval (PFI) [33]. In the same context, low methylation levels of cg11538848 and cg20948051 and high methylation levels of cg23675362, cg18440470, and cg22026687 have been linked to an unfavorable prognosis, thereby highlighting *XPR1* as a potential biomarker with a dual diagnostic and prognostic role in HNSCC [33]. Last but not least, hypomethylation of *CEACAM19* and low expression due to hypermethylation of *RPL29* and *FCGR2C* have been associated with poor survival [53]. Interestingly, an increase in the methylation status of *CEACAM19* (greater than 0.625) has been associated with improved chances of survival [53].

Table 2 includes a list of DNA methylation biomarkers with prognostic potential [24,25,26,28,29,30,31,32,33,34,35,36,37,38,39,40,41,42,43,44,45,46,47,48,49,50,51,52,53,54,55,56,57,58,59,60,61,62,63,64,65,66,67,68,69,70,71,72,73,74,75].

## 4. DNA Methylation Biomarkers with Predictive Potential

Predictive biomarkers provide information on the response (or lack of response) to a specific treatment application and help to personalize therapeutic interventions in patients [19,20].

### 4.1. DNA Methylation Evaluated Pre-Clinically (Cell Lines)

EGFR constitutes one of the most important molecular targets for the treatment of HNSCC [76]. HNSCC cell lines have been investigated with the aim to identify the genes whose different methylation patterns are associated with resistance to anti-EGFR therapies, such as cetuximab and erlotinib [76]. Thus, it has been recognized that hypermethylation of death-associated protein kinase (DAPK) and subsequent transcriptional silencing is associated with resistance to cetuximab and erlotinib. DAPK, therefore, has emerged as a predictive biomarker of anti-EGFR resistance and a potential target for diminishing this type of resistance [76].

In radiation-sensitive cells (SCC-61), DNA methylation appears to be higher, as compared to radiation-resistant cells (Rscc-61), with a total of 84 differentially methylated genes having been identified between the two lines [77]. Related pathways seem to include ILK signaling, glucocorticoid receptor signaling, fatty acid α-oxidation, and cell cycle regulation; among these pathways, the CCND2 protein, which is involved in cell cycle regulation, has been found to exhibit promoter hypermethylation with subsequently reduced expression in Rscc-61 cells, as compared to SCC-61 cells [77]. It has therefore been suggested that CCND2 hypermethylation may be a biomarker of radiation resistance in HNSCC [77]. In parallel, treatment of Rscc-61 and SCC-61 cells with the DNA hypomethylation agent 5-aza-2′deoxycytidine has been shown to induce CCND2 expression levels only in Rscc-61 cells, whereas treatment with the control reagent cytosine arabinoside does not appear to affect gene expression in either cell line [77].

In addition, CD47 overexpression seems to be a prominent feature in radioresistant HNSCC [78]. As CD47 is involved in the molecular pathway that regulates the inhibition of macrophage phagocytosis, it has been suggested that this overexpression probably takes place through the downregulation of tristetraprolin (TTP), which has been shown to inhibit CD47 mRNA degradation in a radioresistant cell line (HN31R), with subsequent inhibition of phagocytosis [78]. It has also been found that TTP downregulation is associated with DNA methylation and overexpression of DNA methyltransferase (DNMT1) [78]. Such observations further suggest that TTP may be involved in the development of radioresistance in HNSCC and that it could constitute a potential biomarker for predicting the efficacy of CD47 antibody-based treatment in recurrent HNSCC following radiotherapy [78].

### 4.2. DNA Methylation Evaluated in Tissue Samples

Cisplatin resistance and its relationship with DNA methylation have been studied in patients with HNSCC, and as a result, six hypermethylated genes have been identified in cisplatin-resistant tumors: *CRIP1*, *G0S2*, *MLH1*, *OPN3*, *S100*, and *TUBB2A* [79]. Interestingly, treatment with the demethylating drug decitabine has been shown to restore the tumor response to cisplatin, while the combination of cisplatin and decitabine appears to decrease tumor development and related pain [79]. Furthermore, the receptor-like PTP type T (*PTPRT*) seems to be frequently hypermethylated in HNSCC; being a negative regulator of *STAT3*, *PTPRT* hypermethylation and subsequent reduction in *PTPRT* expression levels induce an increase in *STAT3* activation and thereby sensitivity to *STAT3* inhibition [80]. These observations indicate that *PTPRT* promoter methylation may serve as a predictive biomarker of response to *STAT3* inhibition and may help to identify patients who would benefit from treatment with *STAT3* targeting agents [80].

Methylation of the PDCD1/PD-1 (programmed cell death protein 1) promoter, in addition to its prognostic role, has also been highlighted for its predictive potential in HNSCC [38]. Different levels of Mpdcd1 have been linked to changes in the lymphocyte compartment, with B lymphocytes infiltrating the tumor and T lymphocytes (CD4+ and CD8+) being inversely correlated with Mpdcd1 [38]. Specifically, methylation of the CD274 promoter (Mcd274) encoding PD-L1 has also been significantly associated with Mpdcd1 and inversely correlated with infiltrating CD8+ T lymphocytes [38]. Therefore, Mpdcd1 could potentially act as a biomarker for predicting the response to immunotherapies targeting the PD-1/PD-L1 axis [38]. Similarly, the predictive role of *OX40* (*TNFRSF4*) and *GITR* (*TNFRSF18*, *AITR*) methylation has been highlighted for use after immunotherapy for HNSCC, in addition to their prognostic role discussed earlier [50]. A positive correlation has been identified between *GITR* and *OX40* mRNA expression with the lymphocyte signature, which in turn is highly related to the leukocyte fraction [50]. Similarly, *OX40* expression has been strongly correlated to the total leukocyte fraction, yet no such correlation has been detected for *GITR* mRNA expression, an observation that is possibly attributed to the differential *GITR* mRNA expression between non-lymphocyte immune cells and lymphocytes [50]. Activated T cells express *OX40*, and this expression is positively correlated with the signatures of Tregs, CD8+ T cells, follicular T helper cells, and active memory CD4+ T lymphocytes, but negatively associated with unknown CD4+ T cells; in contrast, *GITR* mRNA expression has been positively associated with the signatures of Tregs, CD8+ T cells, and follicular T helper cells [50]. Positive correlations have also been noted between *GITR* and *OX40* mRNA expression with signatures of immune cells from the B-cell lineage, with *GITR* expression mostly being associated with memory B cells and plasma cells, and *OX40* expression with naïve B cells [50]. At the same time, interferon-γ signature has been demonstrated to correlate negatively with *GITR* expression and positively with *OX40* expression [50]. The mutational burden has been positively associated with *GITR* mRNA levels and negatively associated with *OX40* transcription levels; it is also weakly and negatively associated with the methylation of CpG11-13, CpG15-16, CpG18, and CpG22 located in the promoter and body of the *GITR* gene, and with the methylation of CpG37 in the promoter of *OX40*, and weakly and positively associated with the methylation of CpG3-5 (downstream of *GITR*), CpG28-29 (transgenic region), CpG33-34 (*OX40* gene body), and CpG43-46 (*OX40* promoter) [50]. In relation to HPV, *OX40* and *GITR* mRNA expression levels appear to be increased in HPV+ tumors [50]. After all these correlations with T-cell infiltration, interferon-γ, HPV status, and mutational burden, it follows that *GITR* and *OX40* methylation and mRNA expression levels can be used as a predictive biomarker for identifying HNSCC patients who would benefit from adjuvant immunotherapy [50].

In an attempt to evaluate the predictive role of methylation for the identification of patients with advanced metastatic HNSCC who would respond to anti-PD-1 immune checkpoint inhibitors (ICIs), >850,000 CpG sites were studied in patients with relapsed or metastatic disease after receiving platinum chemotherapy or who subsequently received anti-PD-1, regardless of their response to the treatment [81]. A methylation signature, including both hypermethylation and hypomethylation profiles, emerged; the genes included in this signature are known to be involved in different molecular pathways, namely axon guidance (e.g., *BMPR1B*, *CAMK2D*, *EPHNA6*, and *NTNG1*), Hippo signaling (e.g., *AFP*, *BMP7*, and *GLI1*), oncogenesis pathways (e.g., *MAPK10*, *IL2RA*, *IGF1R*, *AKT3*, *MLH1*, and *COL4A1*), and MAPK signaling (e.g., *TAOK3*, *STK3*, *IL1RAP*, and *MAP3K1*) [81]. Thus, it has been concluded that the particular DNA methylation signature in HNSCC patients can be used to predict their response to anti-PD-1 immunotherapy [81]. Similarly, the methylation of dual-specificity phosphatase-2 (*DUSP2*) has been investigated in an attempt to predict the response of patients with locally advanced HNSCC (LA-HNSCC) to chemoradiotherapy (CRT) treatment regimens [82]. DUSP2 is a nuclear phosphatase strongly expressed in activated immune cells that catalyzes the dephosphorylation of serine, threonine, and tyrosine residues on different types of mitogen-activated protein kinases inside the MAPK TXY [82]. Considering that both high expression of *EGFR* and *TP53* mutations constitute established markers of malignancy, it has also been noted that patients with low *EGFR* expression and unmethylated *DUSP2* have longer OS compared to patients with low EGFR expression and methylated *DUSP2*, that patients with unmethylated *DUSP2* and *TP53* mutations have better survival outcomes compared to those with wild type *TP53*, and that patients with methylated *DUSP2*, high *EGFR* expression, and wild type *TP53* have the highest OS [82]. All things considered, it has been concluded that the combination of *DUSP2* methylation with *EGFR* expression levels and *TP53* mutation status may represent a biomarker for the prediction of response to CRT in LA-HNSCC [82].

As mentioned earlier, the methylation status of *IDO1* may function as a predictive biomarker [42]. In particular, hypomethylation of the promoter flanking region and the resulting high levels of *IDO1* mRNA expression have shown a positive association with CD8+ and CD4+ T-cell infiltration of the tumor tissue, suggesting that there is a positive feedback mechanism in which in inflammatory T-cell tumors induce the upregulation of *IDO1* [42]. Nonetheless, a negative correlation seems to exist between methylation and tumor mutation burden in all CpG sites [42]. With regards to IFN-γ, a cytokine that is known to induce *IDO1* expression, a positive association has been identified between *IDO1* mRNA expression and all IFN-γ signature genes [42]. Conversely, a negative association has been noted between methylation in the lateral region of the promoter and mRNA expression of IFN-γ signature genes, and a positive association between DNA methylation in the *IDO1* gene body and mRNA expression of IFN-γ signature genes [42]. Based on the above, *IDO1* methylation could serve as a biomarker to predict the response of HNSCC patients to *IDO1* ICIs [42].

Finally, in an attempt to predict the response of HNSCC patients to selective tyrosine kinase inhibitors (TKIs) against the fibroblast growth factor receptor (FGFR) pathway, the methylation profiles of FGFR (FGF1-14, FGF16-23), FGF receptor (FGFR1-4), and cyclin D1 (CCND1) have been investigated for their potential to be used as predictive biomarkers of response to the selective FGFR1/3 inhibitors PD 173074 and AZD4547 [83]. Methylation of five CpGs (CpGs 27, 36, 38, and 42) in the upstream central region of the *CCND1* promoter is significantly correlated with higher ln(IC50) and lower response, respectively, to both inhibitors, with the CpG 36 site showing the strongest effect and the CpG 57 site being negatively correlated with the ln(IC50) of both TKIs. Analysis of the remaining CpG sites has highlighted two sites with significant correlations with the ln(IC50) levels of both inhibitors simultaneously: the CpG 170 site within the *FGFR2* promoter and the CpG 388 site within the *FGF5* promoter [83]. 

Table 3 includes a list of DNA methylation biomarkers with predictive potential [38,42,50,76,77,78,79,80,81,82,83].

**Figure 1:**
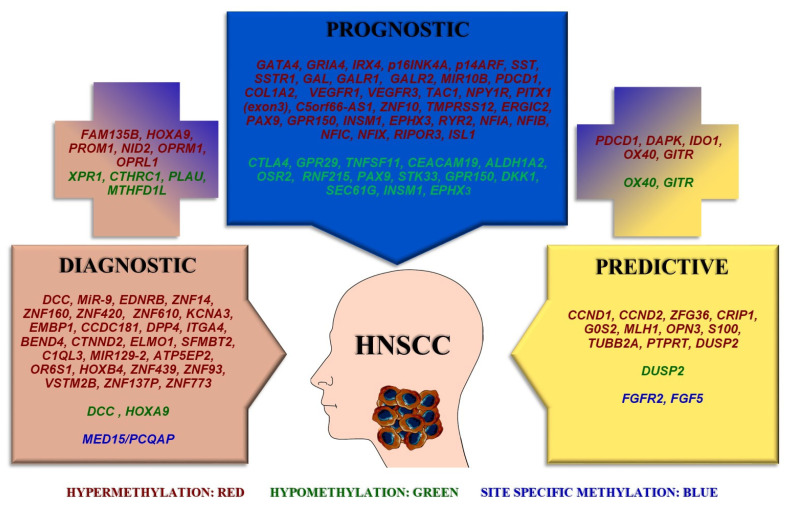
Differentially methylated genes as biomarkers with diagnostic, prognostic, and predictive value in HNSCC. Cancer is a multifactorial disease that depends on the accumulation of both genetic mutations and epigenetic alteration. DNA methylation is one of the major epigenetic mechanisms that enable gene activation or inactivation during tumorigenesis. As such, the methylation profile of patients with HNSCC represents a potential source for new biomarker identification and more ideal patient stratification. Hypermethylation, hypomethylation and site-specific methylation of genes could be utilized as diagnostic, prognostic, and predictive tools. The genes included in the plus signs have dual roles, i.e., either both diagnostic and prognostic (**left**) or both prognostic and predictive (**right**).

Figure 1 summarizes the differentially methylated genes according to their potential to serve as diagnostic, prognostic, or predictive biomarkers.

## 5. Concluding Remarks

This review evaluated gene methylation in patients with HNSCC, with the aim to emphasize the present state of knowledge of DNA methylation as a diagnostic, prognostic, and predictive biomarker.

Regarding diagnostic biomarkers, a total of 13 studies were analyzed [21,22,23,24,25,26,27,28,29,30,31,32,33]. Concerning the type of samples studied, three studies included saliva samples [22,23,24], 12 included tissue samples [21,23,24,25,26,27,28,29,30,31,32,33], and one included plasma samples [32]. In addition, 10 studies focused on HNSCC in general [23,24,25,26,28,29,30,31,32,33], while one study focused mainly on oral and oropharyngeal cancer [21], one concentrated on oral cancer [22], and one on HPV+ oropharyngeal carcinoma [27]. Subsequently, in six studies, the pathological samples showed hypermethylation of the respective gene promoters, with subsequent reduction of transcriptional expression [21,22,25,26,28,32], five studies showed promoter hypomethylation with subsequent increase in transcript levels [25,29,30,31,33], and two studies showed a differential methylation pattern as compared to normal samples [23,24]. Two studies investigated the methylation status of *HOX9*, a transcription factor involved in nuclear maintenance, cell proliferation, cell differentiation, and apoptosis, yet the first of these studies referred to promoter hypermethylation, whereas the second study referred to promoter hypomethylation [25,26]. The other genes found to be differentially methylated include: *miR-9*, the *EDNRB*, *DCC*, *MED15/PCQAP*, *ZNF14*, *ZNF160* and *ZNF420*, *FAM135B*, *ZNF610*, *HOXA9*, *DCC*, *CTHRC*, *PLAU*, *MTHFD1L*, *OPRM1*, *OPRL1*, *XPR1*, *KCNA3*, *EMBP1*, *CCDC181*, *DPP4*, *ITGA4*, *BEND4*, *CTNND2*, *ELMO1*, *SFMBT2*, *C1QL3*, *MIR129-2*, *ATP5EP2*, *OR6S1*, *NID2*, *HOXB4*, *ZNF439*, *ZNF93*, *VSTM2B*, *ZNF137P*, and *ZNF773* [21,22,23,25,26,27,28,29,30,31,32,33]. In addition, a gene signature of five methylated genes, namely *GATA4*, *GRIA4*, *IRX4*, *ALDH1A2*, and *OSR2*, was identified [24].

Regarding the prognostic biomarkers, 29 studies were reviewed [24,25,26,28,29,30,31,32,33,34,35,36,37,38,39,40,41,42,43,44,45,46,47,48,49,50,51,52,53]. The samples were derived from tissues in 29 studies [24,25,26,28,29,30,31,32,33,34,35,36,37,38,39,40,41,42,43,44,45,46,47,48,49,50,51,52,53], while two studies involved blood plasma samples [32,49] and one study involved cell lines [45]. Moreover, 22 studies focused on HNSCC [24,25,26,28,29,30,31,32,33,35,36,38,41,42,44,45,46,47,48,50,53], one study focused mainly on oral and oropharyngeal cancer [33], two on tongue cancer [37,51], two on oral cancer [43,52], one on HPV+ oropharyngeal carcinoma [49], one on HPV− cancer of the hypopharynx, larynx and mouth [39], and one on HPV− HNSCC [40]. Furthermore, hypermethylation was associated with an unfavorable prognosis in 17 studies [26,28,32,34,35,36,38,39,40,41,42,45,46,49,50,51,53], hypermethylation was linked to a favorable prognosis in three studies [25,30,37], hypomethylation was associated with an unfavorable prognosis in nine studies [29,30,31,33,41,44,47,48,53], and hypomethylation was associated with a favorable prognosis in three studies [40,50,51,52]. In total, the following genes were analyzed: methylation signature 1 (*GATA4*, *GRIA4*, *IRX4*, *ALDH1A2*, *OSR2*), *FAM135B*, *HOXA9*, *PROM1/CD133*, *CTHRC1*, *PLAU*, *MTHFD1L*, *OPRM1*, *OPRL1*, *XPR1*, *p16INK4A*, *p14ARF*, *SST*, *SSTR1*, *GAL*, *GALR1/2*, *MIR10B*, methylation signature 2 (*FUT3*, *TRIM5*, *TSPAN7*, *MAP3K8*, *RPS6KA2*, *SLC9A9*, *NPAS3, TIMM8A*, *RNF113A*), *PDCD1*, *COL1A2*, *VEGFR1*, *COL1A2*, *DAPK*, *TAC1*, *GALR1*, *NPY1R*, *SSTR1*, *VEGFR3*, *PITX1*, *C5orf66-AS1* lincRNA gene, methylation signature 3 (*ZNF10*, *TMPRSS12*, *ERGIC2*, *RNF215*), *IDO1*, methylation signature 4 (cg17892178 and cg17378966 in *NID2* and *IDO1*), five DMG mode (*PAX9*, *STK33*, *GPR150*, *INSM1*, *EPHX3*), *RYR2*, *NFI*, *DKK1*, *CALML5*, *DNAJC5G*, *LY6D*, *SEC61G*, *OX40*, *GITR*, *RIPOR3*, *CTLA4*, *GPR29*, *TNFSF11*, *ISL1*, nine-gene multi-omics signature (methylation status of *CEACAM19*, *KRT17*, and *ST18*) [24,25,26,28,29,30,31,32,33,34,35,36,37,38,39,40,41,42,43,44,45,46,47,48,49,50,51,52,53].

Finally, 11 studies evaluated the predictive biomarkers [38,42,50,76,77,78,79,80,81,82,83]. The samples analyzed were derived from tissues [38,42,50,79,80,81,82,83] and cell lines [76,77,78], while all the studies were related to HNSCC in general [38,42,50,76,77,78,79,80,81,82,83]. The therapeutic agents that were tested were immunotherapeutic agents [38,42,50,81], targeted agents [76,80,83], and chemotherapeutic agents [79], as well as radiotherapy [77,78] and chemoradiotherapy [82].

Therefore, during the past decade, a plethora of DNA methylation markers have been identified that show a high level of accuracy and reproducibility in a variety of biospecimens from HNSCC patients that have been obtained with non-invasive or semi-invasive techniques. Epigenetic changes in DNA methylation appear to play an important role in the entire spectrum of HNSCC evolution, from tumor initiation to aggressive disease progression. At the same time, however, there is a need for further identification and stratification of methylation biomarkers, and for the development of robust and efficient detection methods that can be applied to a routine clinical setting of risk assessment, diagnosis (especially at the early stage of the disease), prognosis, treatment management with various therapeutic agents, and post-treatment monitoring.

In general, an epigenetic biomarker should be detectable in the primary tissue or body fluids of the patient, and characterized by stability and reproducibility during sample processing [84]. In order for a specific biomarker to gain approval for clinical application, certain hallmarks should be taken into account [84]. Specifically, the process should include identification of the best genomic regions, pre-analytical processing, and determination of the accuracy of DNA methylation measurements, identification of any confounding parameters, accreditation of the diagnostic procedure, standardized data analysis using the appropriate tools, and of course, specification of the potential costs and patient requirements [84]. In this manner, the technology that enables detection of the biomarker in the relevant clinical material could be developed, first through pre-clinical, analytical validation of the test and then through the development of the standard test [85]. This is a process that requires thorough consideration of the characteristics of the test, such as analytical sensitivity and specificity, accuracy of measurement through clinical sensitivity and specificity, range of measurement, and the definition of the cut-off value for the measurement provided by the test [85]. The prototype must then undergo clinical validation before being promoted commercially as a test [85].

Currently, in vitro methylation-based biomarker diagnostic tests are commercially available for colorectal cancer, glioblastoma, hepatocellular carcinoma, lung and bladder cancer, as well as cervical and prostate cancer [85,86,87]. The technologies used in these tests mainly include standard real-time PCR, real-time PCR with fluorescent hydrolysis probes, methylation-specific real-time PCR, qualitative methylation-specific real-time PCR, quantitative methylation-specific PCR, multiplex real-time PCR, targeted next-generation sequencing, whole-genome bisulfite sequencing, and pyrosequencing; the determination of DNA methylation is performed on fecal samples, blood, plasma, fresh frozen or formalin-fixed, paraffin-embedded tumor tissue, urine, or cervical smear/vaginal specimens/oral scrapes [85,86,87]. In this respect, reverse transcription quantitative real-time PCR (RT-qPCR) systems are emerging as the most versatile systems for DNA methylation analyses [87]. However, there is no equivalent commercially approved test exclusively for head and neck cancer. The pathfinder clinical trial is currently in progress and is focused on evaluating a cell-free DNA-based targeted methylation multi-cancer early detection (MCED) blood testing method that includes head and neck cancer [88].

In conclusion, there is a large chasm between the identification of promising epigenetic signatures and their transfer to clinical practice, even though the number of described epigenetic signatures is rapidly increasing. The adaptation of new methods and technologies can contribute towards the selection of the most valuable epigenetic biomarkers in clinical practice and achieve the transition to precision and personalized medicine.

## Figures and Tables

**Table 1:** Diagnostic and Screening biomarkers.

References	Cancer Type	Biomarker Name-Gene	Function	Sample Type	Biomarker Behavior	Application
[21]	Oral and oropharyngeal	MiR-9	Non-coding RNA implicated in the modulation of gene expression, tumor suppression through inhibition of cellular pro-proliferation and modulation of PTEN.	Tissue	Promoter hypermethylation/sites 1 and 3.Low expression.	Diagnosis
[22]	Oral	EDNRB	It encodes the B-type endothelin receptor (G protein-coupled receptor) that triggers a phosphatidylinositol-calcium second messenger system.	Saliva	Promoter hypermethylation in 40% of malignant samples.	Screening, Diagnosis
[22]	Oral	DCC	Tumor suppressor, encodes a transmembrane protein with structural homology to NCAM, which is associated with the differentiation of epithelial and neuronal cells.	Saliva	Promoter hypermethylation in 40% of malignant samples.	Screening, Diagnosis
[23]	HNSCC	MED15/ PCQAP	Encodes a cofactor with pleiotropic activity, important for the formation of the RNA polymerase II complex, which is involved in the expression of all protein-coding genes, attenuation of at least one of the signaling pathways (TGFβ/Activin signaling).	Tissue, saliva	Specific methylation patterns in the promoter.	Screening, Diagnosis
[24]	HNSCC	ZNF14, ZNF160, ZNF420	Members of the zinc finger gene family.	Tissue, saliva	Differential methylation pattern.	Diagnosis
[25]	HNSCC	FAM135B	Involved in the maintenance of the nucleolus, cell proliferation, cell differentiation, and apoptosis.	Tissue	Hypermethylation. Low expression.	Diagnosis
[25]	HNSCC	ZNF610	Involved in the maintenance of the nucleolus, cell proliferation, cell differentiation, and apoptosis.	Tissue	Hypermethylation. Low expression.	Diagnosis
[25]	HNSCC	HOXA9	Involved in the maintenance of the nucleolus, cell proliferation, cell differentiation, and apoptosis.	Tissue	Hypomethylation. High expression.	Diagnosis
[25]	HNSCC	DCC	Involved in the maintenance of the nucleolus, cell proliferation, cell differentiation, and apoptosis.	Tissue	Hypomethylation. High expression.	Diagnosis
[26]	HNSCC	HOXA9	Encodes a transcription factor. HOX genes regulate and specify different cell types during embryonic growth, and have important functions in the modulation of the sensitive balance between cell proliferation and differentiation during cancer development.	Tissue	Promoter hypermethylation.	Screening, Diagnosis
[27]	HPV+ OPSCC	KCNA3, EMBP1, CCDC181, DPP4, ITGA4, BEND4, CTNND2, ELMO1, SFMBT2, C1QL3, MIR129-2, ATP5EP2, OR6S1, NID2, HOXB4, ZNF439, ZNF93, VSTM2B, ZNF137P and ZNF773	ITGA4: on the cell surface, promotes migration and adhesion to the microenvironment in chronic lymphocytic leukemia. NID2: component of the basement membrane that stabilizes the extracellular matrix (ECM) network. Suppresses migration and blocks metastasis by downregulating the EGFR/AKT and integrin/FAK/PLCγ pathways. HOXB4: a hematopoietic transcription factor, downregulates the expression of Prdm16, which is a proto-oncogene necessary for self-renewal and preservation of mouse hematopoietic stem cells. SFMBT2: tumor suppressor, negatively regulates migration and invasion by targeting MMP-9 and MMP-26. MIR129-2: tumor suppressor, inhibits migration and invasion by directly inhibiting HMGB1. DPP4 and CTNND2: act both as tumor suppressors and as markers of tumor invasiveness, depending on the type of tumor. KCNA3: its knockdown markedly suppresses cell proliferation and increases apoptosis. EMBP1: related to ER-positive breast cancer and lower grade breast tumors. ZNF93: implicated in the DNA repair pathway following DNA damage by chemotherapy. Little is known about six candidates (ATP5EP2, OR6S1, ZNF439, VSTM2B, ZNF137P, ZNF773). ZNF439, ZNF137P, ZNF773: belong to the zinc finger protein group, members of which have previously been shown to have tumor-suppressive activity.	Tissue	20 differentially DMRs → hypermethylation.	Screening
[28]	HNSCC	PROM1/CD133	Encodes a pentaspan membrane glycoprotein, which is frequently found in adult stem cells.	Tissue	Promoter hypermethylation. Low expression.	Diagnosis
[29]	HNSCC	CTHRC1	Encodes an extracellular matrix protein, modulating tumor microenvironment	Tissue	Promoter hypomethylation. High expression.	Diagnosis
[30]	HNSCC	PLAU	Encodes a protease implicated in apoptosis, epithelial-mesenchymal transition (EMT), and the Ras/MAPK pathway.	Tissue	Hypomethylation. High expression.	Diagnosis
[31]	HNSCC	MTHFD1L	Encodes a cytoplasmic enzyme participating in the formation of tetrahydrofolic acid (THF) within mitochondria.	Tissue	Promoter hypomethylation. High expression.	Diagnosis
[32]	HNSCC	Opioid receptor mu 1(OPRM1)	Encodes an opioid receptor (G-protein-coupled receptor).	Tissue, plasma	Hypermethylation.	Screening
[32]	HNSCC	Opioid-related nociceptin receptor 1(OPRL1)	Encodes an opioid receptor (G-protein-coupled receptor).	Tissue, plasma	Hypermethylation.	Screening
[33]	HNSCC	XPR1	Encodes a cell surface receptor for certain types of murine leukemia viruses, which plays an essential role in maintaining intracellular phosphate homeostasis by mediating phosphate export from the cell.	Tissue	Promoter hypomethylation. High expression.	Diagnosis

**Table 2:** Prognostic biomarkers.

References	Cancer Type	Biomarker Name-Gene	Function	Sample Type	Biomarker Behavior	Application
[24]	HNSCC	Methylation signature of the promoter of 5 genes, GATA4, GRIA4, IRX4 ALDH1A2, OSR2	GATA4: encodes GATA binding protein 4, a member of the GATA family of zinc-finger transcription factors. GRIA4: encodes a glutamate receptor. IRX4: encodes Iroquois homeobox 4. ALDH1A2: encodes aldehyde dehydrogenase 1 family member A2, an enzyme that catalyzes the synthesis of retinoic acid from retinaldehyde. OSR2: odd-skipped related transcription factor 2.	Tissue	GATA4, GRIA4, IRX4: promoter hypermethylation. ALDH1A2 and OSR2: promoter hypomethylation.	Prognosis
[25]	HNSCC	FAM135B	Involved in the maintenance of the nucleolus, cell proliferation, cell differentiation, and apoptosis.	Tissue	hypermethylation → favorable prognostic feature	Prognosis
[26]	HNSCC	HOXA9	Encodes a transcription factor involved in the maintenance of the nucleolus, in cell proliferation and differentiation, and in apoptosis.	Tissue	Promoter hypermethylation → progression and metastasis.	Prognosis
[28]	HNSCC	PROM1/ CD133	Encodes a pentaspan membrane glycoprotein, often expressed on adult stem cells.	Tissue	Promoter hypermethylation-low expression → poor overall survival and relapse-free survival.	Prognosis
[29]	HNSCC	CTHRC1	Encodes an extracellular matrix protein, involved in the modulating of the tumor microenvironment.	Tissue	Overexpression → decreased overall survival (OS).	Prognosis
[30]	HNSCC	PLAU	Encodes a protease, involved in apoptosis, epithelial–mesenchymal transition (EMT), and Ras/MAPK pathway.	Tissue	Hypomethylation → higher clinical stage, more aggressive metastatic disease. Hypermethylation → increased survival time.	Prognosis
[31]	HNSCC	MTHFD1L	Encodes a cytoplasmic enzyme, involved in the formation of tetrahydrofolate (THF) within mitochondria.	Tissue	High expression → decreased OS.	Prognosis
[32]	HNSCC	opioid receptor mu 1(OPRM1)	Encodes an opioid receptor (G-protein-coupled receptor).	Tissue, plasma	Hypermethylation → aggressive clinical behavior.	Prognosis
[32]	HNSCC	opioid-related nociceptin receptor 1(OPRL1)	Encodes an opioid receptor (G-protein-coupled receptor).	Tissue, plasma	Hypermethylation → aggressive clinical behavior.	Prognosis
[33]	HNSCC	XPR1	Encodes a cell surface receptor for certain types of murine leukemia viruses that is involved in the maintenance of the intracellular phosphate homeostasis by mediating phosphate export from the cell.	Tissue	High expression → lower OS, DSS and PFI.	Prognosis
[34]	Oral and oropharyngeal squamous cell carcinoma (OSCC)	p16INK4A p14ARF	Tumor suppressors, encode cell cycle regulatory proteins.	Tissue	Promoter hypermethylation-low expression. P16 promoter hypermethylation → progression of premalignant lesions to OSCC, advanced disease, local recurrence, and lower DSS (disease-specific survival).	Prognosis
[35]	HNSCC	SST	Tumor suppressor, encodes a growth hormone release-inhibitory factor.	Tissue	Promoter hypermethylation-low expression. Hypermethylation → decreased disease-free survival, tumor recurrence after resection.	Prognosis
[35]	HNSCC	SSTR1	Tumor suppressor, encodes a somatostatin receptor type 1.	Tissue	Promoter hypermethylation-low expression. Hypermethylation → decreased disease-free survival, tumor recurrence after resection.	Prognosis
[36]	HNSCC	GAL	Tumor suppressor, encodes a 30-amino acid peptide in humans, that targets the galanin system via receptors GALR1, GALR2, and GALR3.	Tissue	Promoter hypermethylation-low expression. Hypermethylation → reduced disease-free survival.	Prognosis
[36]	HNSCC	GALR1/2	Tumor suppressors, encode galanin receptors (G protein-coupled receptors).	Tissue	Promoter hypermethylation-low expression. Hypermethylation → reduced disease-free survival.	Prognosis
[37]	OTSCC	MIR10B	Encodes a miRNA that targets oncogenes NR4A3 and BCL2L11.	Tissue	Hypermethylation-low expression → downregulation of NR4A3 and BCL2L11 → better disease-free survival.	Prognosis
[37]	OTSCC	FUT3, TRIM5, TSPAN7, MAP3K8, RPS6KA2, SLC9A9, NPAS3 TIMM8A, RNF113A genes	FUT3: regulates the expression of Lewis antigens in the human Lewis blood group system [54]. TRIM5: acts as a selective autophagy receptor that targets autophagic substrates via direct recognition [55]. TSPAN7: cell surface glycoprotein and may have a role in the control of neurite outgrowth, synaptic transmission, and viral-induced inflammation [56]. MAP3K8: proto-oncogene that participates in the MAP kinase and JNK signaling pathways [57]. RPS6KA2: controls cell growth and differentiation [58]. SLC9A9: autism-risk gene localized in the endosomal system that is involved in the endocytic regulation of autophagy and cell survival [59]. NPAS3: encodes a transcription factor that is expressed in the developing central nervous system [61]. TIMM8A: localizes to the intermembrane space in mitochondria where it functions in the import of nuclear-encoded proteins into the mitochondrial inner membrane [60]. RNF113A: involved in gene regulation and DNA repair [62].	Tissue	Methylation signature, differential methylation among the various categories of parameters.	Prognosis
[38]	HNSCC	PDCD1 (PD-1)	Programmed cell death protein 1, immune inhibitory receptor (also known as CD279 or PDCD1), a member of the extended CD28/CTLA-4 family, it is stably expressed only on T cells exposed to a chronic antigen.	Tissue	Hypermethylation → shorter OS after surgical resection.	Prognosis
[39]	HPV negative hypopharyngeal cancer	COL1A2	Encodes a fibrillar collagen found in most connective tissues and is the main component of the organic part of bones.	Tissue	Hypermethylation-low expression → reduced survival and a higher likelihood of relapse.	Prognosis
[39]	HPV negative laryngeal cancer	COL1A2	Encodes a fibrillar collagen found in most connective tissues and is the main component of the organic part of bones.	Tissue	Hypermethylation-low expression → worse survival.	Prognosis
[39]	HPV negative laryngeal cancer	p16, VEGFR1, VEGFR3, DAPK, TAC1, GALR1, NPY1R, SSTR1	p16: tumor suppressor gene that prevents oncogenic transformation through the induction of cellular senescence [63]. VEGFR1: stimulates angiogenesis and vascular permeability [64]. VEGFR3: regulates the development and maintenance of the lymphatic system [65]. DAPK: involved in multiple cellular signaling pathways that trigger autophagy, apoptosis, and cell survival [66]. TAC1: functions as a neurotransmitter interacting with both nerve receptors and smooth muscle cells [67]. GALR1: acts as a neuropeptide that modulating various physiological functions, including nociception, cognition, and neuroendocrine regulation [68]. NPY1R: acts as a neuropeptide that regulates several physiological processes including food intake, emotional regulation, and cardiovascular function [69]. SSTR1: neuropeptide that is involved in neurotransmission, secretion, and cell proliferation [70].	Tissue	Hypermethylation-low expression → worse survival, higher risk of recurrence.	Prognosis
[40]	HNSCC, especially in HPV-negative	PITX1 3 exon	Encodes a transcription factor in embryogenesis that is involved in mouth and hindlimb formation and pituitary development, it is an upstream inducer of RASAL1 and therefore an important mediator of the Ras signaling pathway.	Tissue	Hypermethylation-low expression → increased risk for death.	Prognosis
[40]	HNSCC, especially in HPV-negative	C5orf66-AS1 lincRNA	Encodes a long intergenic non-coding RNA, involved in tumorigenesis, likely by acting as (post)-transcriptional regulator of PITX1.	Tissue	Hypermethylation-low expression → increased risk for death. Hypomethylation → better overall survival.	Prognosis
[41]	HNSCC	Methylation signature of ZNF10 TMPRSS12 ERGIC2 RNF215	ZNF10: encodes a transcription repressor, involved in development, differentiation, and metabolism. TMPRSS12: encodes a member of the serine protease family that participates in immune response and blood coagulation and production, it is associated with human infertility. ERGIC2 protein: encodes an endoplasmic reticulum (ER) resident protein implicated in protein trafficking between ER and Golgi bodies. RNF125 gene: encodes a novel E3 ubiquitin-protein ligase that may be involved in the T-cell receptor signaling pathway, plays a role in tumorigenesis and metastasis, strengthens p53 degradation, and suppresses p53 function.	Tissue	Hypermethylation of ZNF10 as well as TMPRSS12 and ERGIC2 → unfavorable prognosis. Hypomethylation-high expression of RNF125 → poorer overall survival.	Prognosis
[42]	HNSCC	IDO1	Encodes an indoleamine 2,3-dioxygenase (IDO)—an enzyme that limits the rate of conversion of the crucial amino acid tryptophan to kynurenine, it is strongly expressed in many types of tumors and has been shown to play a role in immunosuppression through increased tryptophan metabolism in the tumor microenvironment (TME). Increased expression of IDO1 can result in suppression of anti-tumor T-cells, differentiation of CD4+ T-cells into immunosuppressive regulatory T-cells (Tregs), and polarization of anti-gene cells into a tolerogenic phenotype.	Tissue	Hypermethylation of the lateral promoter → poor overall survival.	Prognosis,
[43]	Oral squamous cell carcinoma (OSCC)	Methylation signature of cg17892178 and cg17378966 in NID2 and IDO1	NID2: encodes for a cell adhesion protein involved in maintaining the structure of the vascular extracellular matrix [71]. IDO1: rate-limiting enzyme involved in tryptophan catabolism; also acts as an immune checkpoint molecule on the surface of dendritic cells [72].	Tissue	AUC = 0.81 indicated that the signature prognostic risk score based on two CpGs was a good prognostic factor of survival in patients with OSCC.	Prognosis
[44]	HNSCC	five DMG model PAX9, STK33, GPR150, INSM1, and EPHX3	Encode genes that are involved in extracellular matrix, cell adhesion, and immune responses.	Tissue	Hypomethylation of PAX9, STK33, GPR150, INSM1, and EPHX3 → lower survival time and increased tumor-related mortality PAX9, GPR150, INSM1, and EPHX3 with hypermethylation and weak expression and hypomethylation and elevated expression → strongly associated with OS.	Prognosis
[45]	HNSCC	RYR2	Encodes an important component of the intracellular Ca^2+^ release pathway, it is associated with the sarcoplasmic or endoplasmic reticulum of various cell types, particularly in cardiomyocytes.	Tissue, cell lines	Variable methylation profile. Gradual promoter hypermethylation-low expression → upcoming cancerous transformation of dysplastic lesions.	Prognosis
[46]	HNSCC	nuclear factor I (NFI): NFIA, NFIB, NFIC and NFIX	Transcription factors	Tissue	Promoter hypermethylation-low expression. Decreased levels of NFIA, NFIB, and NFIC → shorter overall survival.	Prognosis
[47]	HNSCC	DKK1	Encodes a protein with inhibitory activity on the Wnt signaling pathway.	Tissue	Promoter hypomethylation-high expression. Overexpression → worse OS-DFS.	Prognosis
[49]	HPV+ OPC	CALML5	Encodes a skin-specific calcium-binding protein, which is strongly related to the differentiation of keratinocytes. It is a regulator of the final differentiation of epidermal cells and CALML5 high-density conditions promote the translocation of YAP1 into the cytoplasm.	Tissue, plasma	Methylation → shorter DFS.	Prognosis
[49]	HPV+ OPC	DNAJC5G	Encodes a protein that inhibits the replication efficiencies of adenovirus, vaccinia virus, and HIV-1.	Tissue, plasma	Methylation → shorter DFS.	Prognosis
[49]	HPV+ OPC	LY6D	Encodes a membrane-bound protein with a glycosylphosphatidylinositol anchor. It has an important role in the adhesion of head and neck cancer cells to endothelial cells, and is detected of micrometastases in lymph nodes of HNSCC patients.	Tissue, plasma	Methylation → shorter DFS.	Prognosis
[48]	HNSCC	SEC61G	Encodes a transmembrane heterotrimeric channel protein that transports nascent polypeptides and proteins to the ER, mediating membrane protein degradation.	Tissue	Hypomethylation-high expression. Overexpression → worse OS.	Prognosis
[50]	HNSCC	OX40 (TNFRSF4)	Encodes a tumor necrosis factor receptor, which is expressed mainly on the surface of activated T cells and is stimulated by the OX40L ligand, which is found on antigen-presenting cells, activated T cells, lymphoid tissue inductor cells, some endothelial cells, and mast cells. Its up-regulation promotes differentiation, proliferation, and prolonged survival of T cell activation through inhibition of activation-induced cell death as well as stimulation of cytokine production.	Tissue	Promoter hypermethylation → worse overall survival. Higher expression levels → longer overall survival.	Prognosis,
[50]	HNSCC	GITR (TNFRSF18, AITR)	Encodes a tumor necrosis factor receptor, expressed at high levels by regulatory T cells (Tregs) and at lower levels by naïve, effector and memory T cells. The binding of the GITR ligand in conjunction with T cell receptor stimulation induces activation of the MAPK/ERK and NFkB pathway, resulting in immunoregulation of the immune system with T cell proliferation, production of proinflammatory cytokines, enhanced anti-cancer effector function and resistance of CD4+ and CD8+ T cells. GITR promotes anti-cancer immunity by enhancing effector T cell function on the one hand and inhibiting Treg proliferation.	Tissue	Promoter hypermethylation → worse overall survival. Higher expression levels → longer overall survival.	Prognosis,
[51]	Oral Tongue Cancer	RIPOR3	Encodes a member of the extracellular region, regulates cellular processes in response to stimuli that is associated with immune cell infiltrations.	Tissue	Hypermethylation-low expression. Hypermethylation-low expression → poor prognosis. High expression → higher OS, PFS.	Prognosis
[52]	OSCC	CTLA4 (Cytotoxic T-lymphocyte associated protein 4)	Encodes a protein that is expressed in activated CD4+ T cells and constitutively expressed in CD4+Foxp3+ Treg cells. It is a potent immune inhibitor by decreasing the onset of T-cell activation mediated by the interactions between antigen presenting cells (APCs) and T cells.	Tissue	Hypomethylation. High expression → higher long-term survival.	Prognosis
[52]	OSCC	GPR29	Encodes a G protein-coupled receptor. It is a chemokine receptor that is primarily expressed by T cells, immature dendritic cells, and B cells. Its unique partner CCL20 is known to be stably expressed by Th17 cells and secreted by intestinal epithelial cells in response to local enteropathogenic infection. The CCL20-CCR6 axis has a critical role in mucosal immunity, while the increased expression of CCR6 has previously been demonstrated in several cancers, with complicated anti-cancer or pre-cancer potential.	Tissue	Hypomethylation. High expression → higher long-term survival.	Prognosis
[52]	OSCC	TNFSF11	Encodes a receptor activator of nuclear factor receptor kappa-B ligand (RANKL). It binds to the RANK receptor to modulate differentiation, activation, and survival of osteoclast cells. It has also been implicated in pathways such as the innate immune response, cell proliferation, and apoptosis.	Tissue	Hypomethylation. High expression → higher long-term survival.	Prognosis
[52]	OSCC	ISL1(ISL LIM homeobox 1)	Encodes the LIM-homeodomain transcription factor, which has been involved in many significant biological pathways, such as carcinogenesis, cell invasion, apoptosis, and cancer immunity.	Tissue	Hypermethylation. High expression → higher long-term survival.	Prognosis
[53]	HNSCC	Nine-gene multi-omics signature (methylation status of CEACAM19, KRT17, and ST18)	CEACAM19: immunoglobulin gene encoding for a variety of glycoproteins that are implicated in embryonic development, immunity, and may also serve as receptors for viruses and bacteria [73]. KRT17: involved in the formation and maintenance of several types of skin appendages, specifically in the shape and orientation of hair [74]. ST18: neural zinc finger transcription factor that negatively regulates cell proliferation [75].	Tissue	Absence of high methylation of CEACAM19 → poor survival. Low expression of RPL29 and FCGR2C → poor survival. A methylation status greater than 0.625 in CEACAM19 → greater probability of survival.	Prognosis

**Table 3:** Predictive biomarkers.

References	Cancer Type	Biomarker Name-Gene	Function	Sample Type	Biomarker Behavior	Application
[38]	HNSCC	PDCD1 (PD-1)	The PD1/PD-L1 axis is implicated in the downregulation of immune responses in tumors, affecting T-cell responses in secondary lymphoid tissues, moving the balance from T-cell activation to antigen tolerance.	Tissue	Mpdcd1 → response to immunotherapies targeting the PD-1/PD-L1 axis.	Prediction
[42]	HNSCC	IDO1	Encodes indoleamine 2,3-dioxygenase (IDO)—an enzyme that limits the rate of conversion of the crucial amino acid tryptophan to kynurenine, it is strongly expressed in many types of tumors and has been shown to play a role in the immunosuppression of naïve cells through increased tryptophan metabolism in the tumor microenvironment (TME). Increased expression of IDO1 can result in suppression of anti-tumor T-cells, differentiation of CD4+ T-cells into immunosuppressive regulatory T-cells (Tregs), and polarization of anti-gene cells into a tolerogenic phenotype.	Tissue	Response to IDO1 immune checkpoint inhibitors.	Prediction
[50]	HNSCC	OX40 (TNFRSF4)	Encodes tumor necrosis factor receptor, which is primarily expressed on the surface of activated T cells and is stimulated by its receptor OX40L, which is located on antigenic cells, activated T cells, lymphoid tissue inductor cells, certain endothelial cells, and mast cells. Its regulation induces differentiation, proliferation, and extended survival of T cell-activated cells through inhibition of activation-induced cell death, as well as stimulation of cytokine synthesis.	Tissue	Identifying HNSCC patients who would benefit from adjuvant immunotherapy.	Prediction
[50]	HNSCC	GITR (TNFRSF18, AITR)	Encodes the tumor necrosis factor receptor, is highly expressed by regulatory T cells (Tregs) and expressed at lower levels on naïve, effector, and memory T cells. Binding of the GITR ligand in combination with T cell receptor stimulation causes activation of the MAPK/ERK and NFkB pathway, resulting in immune system upregulation with T cell proliferation, production of proinflammatory cytokines, enhanced anti-cancer effector function, and resistance of CD4+ and CD8+ T cells. GITR promotes anti-cancer immunity by enhancing effector T cell function and by suppressing Treg proliferation.	Tissue	Identifying HNSCC patients who would benefit from adjuvant immunotherapy.	Prediction
[76]	HNSCC	DAPK	Encodes calcium/calmodulin (CaM)-regulated serine/threonine protein kinase, has pro-apoptotic function, mediates cell death triggered by a variety of death-inducers, including interferon-γ, 20 TGF, 21 TNFα, and Fas ligand.	Cell lines	Hypermethylation -low expression → resistance to cetuximab and erlotinib.	Prediction
[77]	HNSCC	DNA methylation	ILK signaling, glucocorticoid receptor signaling, fatty acid α-oxidation, cell cycle regulation.	Cell lines	Hypermethylation → radiation resistance.	Prediction
[78]	HNSCC	ZFG36 gene -TTP	Tumor-suppressor, encodes Arna-binding protein, enhances decay of AU-rich element (ARE)-containing transcripts, and plays an important role in cellular differentiation, proliferation, tumorigenesis, and immunity, modulates the posttranscriptional control of inflammatory mediators, and immune gene expression.	Cell lines	Hypermethylation-low expression → overexpression of CD47 in a radioresistant cell line (HN31R) → inhibition of phagocytosis. Prediction of the efficacy of CD47 antibody in recurrent HNSCC patients after radiotherapy.	Prediction
[79]	HNSCC	Gene panel (CRIP1, G0S2, MLH1, OPN3, S100 and TUBB2A)	CRIP1: Cysteine Rich Protein 1 G0S2: G0/G1 switch gene 2 Mlh1: MutL protein homolog 1 OPN3: Opsin 3 S100 TUBB2A: Tubulin Beta 2A Class Iia	Tissue	Hypermethylation → cisplatin-resistance.	Prediction
[80]	HNSCC	PTPRT	A member of the PTPR family, receptor of PTPs (enzymes that catalyze the removal of a phosphate group from specific signaling proteins), they cover the membrane once and contain one or two intracellular catalytic sites, as well as a modular extracellular region that typically contains several protein-protein interaction sites.	Tissue	Hypermethylation → sensitivity to STAT3 targeting agents.	Prediction
[81]	HNSCC	DNA methylation profile	Genes involved in different molecular pathways, namely Axon guidance, Hippo signaling, Pathways in cancer and MAPK signaling	Tissue	Both hypermethylation and hypomethylation → predict response to ICI.	Prediction
[82]	LA-HNSCC	DUSP2	Encodes a nuclear phosphatase that is strongly expressed in activated immune cells and catalyzes the dephosphorylation of serine, threonine, and tyrosine residues on various types of mitogen-activated protein kinases inside MAPK TXY.	Tissue	Patients treated with CRT: Low EGFR + unmethylated DUSP2 → longer overall survival Compared to low EGFR + meth-DUSP2 Unmethylated DUSP2 + Mtp53 → longer survival compared to unmethylated DUSP2 + wtTP53. Methylated DUSP2 + high EGFR, + wild type TP53 → highest OS and HR.	Prediction
[83]	HNSCC	CCND1	Encodes a regulator of the G1/S phase transition, it is degraded as the cell enters the S phase.	Tissue	Response to selective FGFR1/3 inhibitors PD 173074 and AZD4547.	Prediction
[83]	HNSCC	FGFR2	Encodes a fibroblast growth factor receptor.	Tissue	Response to selective FGFR1/3 inhibitors PD 173074 and AZD4547.	Prediction
[83]	HNSCC	FGF5	Encodes a cell signaling protein.	Tissue	Response to selective FGFR1/3 inhibitors PD 173074 and AZD4547.	Prediction

## Data Availability

Not applicable.
